# The re-emergence of dengue in China

**DOI:** 10.1186/s12916-015-0345-0

**Published:** 2015-04-28

**Authors:** Eng Eong Ooi

**Affiliations:** Program in Emerging Infectious Diseases, Duke-NUS Graduate Medical School, 8 College Road, Singapore, 169857 Singapore

**Keywords:** Dengue, Epidemic, Surveillance, Re-emergence, China

## Abstract

The number of reports in the literature on dengue outbreaks in various parts of south China is increasing. This trend is likely contributed to by multiple factors, chief among which is the increase in trade and human movement in and out of China from the Southeast Asian region where dengue is firmly endemic. However, a holistic picture of dengue in China and how the public health authorities are responding to this global health challenge has been missing. In a research article published in *BMC Medicine*, Lai *et al.* have now filled this gap in knowledge by analysing statutorily mandated national dengue surveillance data from 1990 till 2014. They also conducted time series analyses to identify key drivers of dengue transmission in south China as well as from south China to the other parts of this vast and populous country. Their findings, as well as the description of surveillance and disease control activities in China, highlight urgent steps that need to be taken if China wishes to prevent itself from becoming another country that experiences large and frequent cycles of epidemic dengue.

Please see related article: http://dx.doi.org/10.1186/s12916-015-0336-1.

## Background

Dengue in China has a long history. Clinical description consistent with dengue was recorded during the Jin Dynasty in A.D. 265 to 420 and one of the earliest dengue epidemics occurred in A.D. 992, both in China [1]. The emergence of three- to five-year cycles of epidemic dengue in the latter half of the 20^th^ century has, however, spared China. Indeed, the epicentre of the emergence of cyclical dengue epidemics began in Southeast Asia, immediately south of China, after World War II (WWII). The movement of troops and war materiel introduced both the vector, *Aedes aegypti,* and the dengue virus (DENV) into this region of the world. The post war recovery resulted in intensive urbanisation in cities such as Manila, Bangkok and Kuala Lumpur. Urbanisation allowed the highly domesticated *A. aegypti* mosquito to thrive and the increasing human population density provided susceptible hosts for all four serotypes of DENV [2]. From this foothold and starting in the 1950s, cyclical epidemic dengue spread to the rest of the tropical and subtropical world, except, perhaps, China.

China borders several Southeast Asian countries, namely Vietnam, Laos and Myanmar. It also borders Bangladesh and India, where dengue is endemic. South China has a humid subtropical climate and is on the same latitude as south Taiwan and Mexico, where repeated dengue epidemics have been reported. South China also has a number of densely populated urban centres, such as Guangdong and Shenzhen. Despite these favourable contexts for dengue transmission, dengue cases and epidemics have only been reported sporadically in China, until now.

In a research article in *BMC Medicine*, Lai *et al.* provide a comprehensive description of dengue incidence in China [3]. Dengue became a legally mandated notifiable disease in China in 1989 after its re-emergence in 1978. Lai and colleagues obtained and analysed all notified cases of dengue from 1 January 1990 to 31 December 2014. They show that the average annual incidence rate of dengue is 2.2 cases per million persons, although the incidence showed an increasing trend since 2012, peaking in 2014 with a record high of over 34.4 cases per million persons. Time series analyses of the reported dengue cases indicated that indigenous cases occurred mostly between July and November. These indigenous cases were often preceded by reports of imported dengue cases. As expected, the provinces with the highest incidence of dengue were those that border Southeast Asian countries. All four DENV serotypes have been detected, some of which have also spread northwards and westwards in China. The trends since 2012 could thus be a harbinger of cyclical epidemic dengue in China.

### Why is epidemic dengue a relatively latecomer in China despite its proximity to Southeast Asia?

For many years post WWII, international trade and travel to and from China were limited due to economic ideals and policies instituted under the Chairmanship of Mao. Besides economic isolation, these policies probably also served to limit the introduction of DENV through asymptomatic or pre-symptomatic travellers into China via the big Southeast Asian cities. The gradual opening up of China economically under the leadership of Deng Xiaoping, from 1978 until 1992 led to more open trade and travel [4] and this policy continues to be in place presently. It is thus not completely surprising that outbreaks occurred from 1978 onwards. However, it has also to be said that the lack of reports of disease does not invariably mean low disease incidence. The types, quality and intensity of surveillance activities are also critical factors and these will be discussed later.

### What are the implications of re-emergence of dengue in China?

South China has several large cities. Guangzhou and Shenzhen each has more than 10 million inhabitants. These cities and many more are growing economically and the size and population densities are set for expansion. These factors could provide environments that are even more conducive for DENV transmission and, hence, more frequent and larger epidemics can be expected in the coming years. Instead of merely being on the receiving end of imported dengue, south China could become a nidus of dengue transmission, not only to other parts of China but also to the rest of the world.

### What then should our response be?

Besides describing the scale of the problem, Lai and colleagues also highlight areas in disease and vector surveillance for improvement [3]. Dengue surveillance in China, as in many countries, is passive. This likely contributes to significant underestimation of the true burden of disease. Given the non-specific clinical features of dengue, especially in the early phase of illness, a more active approach to dengue surveillance will be needed to obtain a more accurate epidemiological picture [5]. Indeed, the low incidence of dengue in the 1990s despite the use of clinical diagnostic criteria alone suggests that awareness of dengue may not be high. The use of clinical diagnosis without laboratory confirmation would produce a high sensitivity but poor specificity for acute dengue [6] and this should have led to over-diagnosis of dengue until the introduction of laboratory confirmatory tests. That this trend did not occur underscores the need for a greater level of outreach by the public health authorities to physicians working at all levels of healthcare to raise awareness. Likewise, the use of active surveillance in selected locales could also provide more refined data on the true burden of dengue in China [3].

Unhelpful criteria for classifying dengue should also be removed. A major problem is the requirement of a recall of mosquito bite within 15 days of illness onset. This requirement likely introduces recall bias into the surveillance data. Moreover, the night time buzz or bites of mosquitoes that then wake a person are more likely to be remembered than those that occur during the daytime, which is when the peak biting activity of *A. aegypti* and *A. albopictus* occurs. This inclusion criterion thus introduces unnecessary inaccuracies into the surveillance data.

Finally, the scale of vector surveillance and population control will also need to be ramped up significantly. Lai *et al.* noted that out of 483 counties in the five provinces in south China, only 16 conducted vector surveillance. This is clearly insufficient to prevent dengue. The authors also reported that only one province, Hainan, is infested with *A. aegypti*. All others have only reported *A. albopictus* infestation. It is plausible that, with the limited surveillance, the geographic spread of *A. aegypti*, which display biological and behavioral features that make it the more effective DENV vector [7], is currently underestimated. Furthermore, without systematic surveillance, the spread of *A. aegypti* through the movement of goods and other materials into new parts of China would also go undetected until a dengue epidemic is well underway. When this happens, any emergency vector control response would often be too little too late. Given the scale of expansion of the highly urbanised and populated areas in south China, steps to rectify this gap in surveillance should be taken urgently.
